# New peroral pancreatoscopy for direct diagnosis and precise guidewire placement in indeterminate pancreatic duct stricture

**DOI:** 10.1055/a-2660-3552

**Published:** 2025-08-20

**Authors:** Meijuan Zhang, Yawen Liang, Shengxin Chen, Ke Meng, Baoguo Bu, Mingyang Li, Yaqi Zhai

**Affiliations:** 1 651943 Division of Gastroenterology and Hepatology, The First Medical Center, Chinese PLA General Hospital, Beijing, China; 2 636787 Shanxi Provincial Hospital of Traditional Chinese Medicine, Shaanxi, China

A 71-year-old woman was hospitalized for a main pancreatic duct (MPD) stricture of unknown origin. She was asymptomatic and had a history of cholelithiasis with endoscopic retrograde cholangiopancreatography (ERCP) and following cholecystectomy, yet she denied any history of pancreatitis. Laboratory tests revealed no significant abnormalities. The computed tomography and magnetic resonance cholangiopancreatography showed the short-segment MPD stricture in the body without a definite pancreatic mass ( Fig. 1 ).

**Fig. 1:**
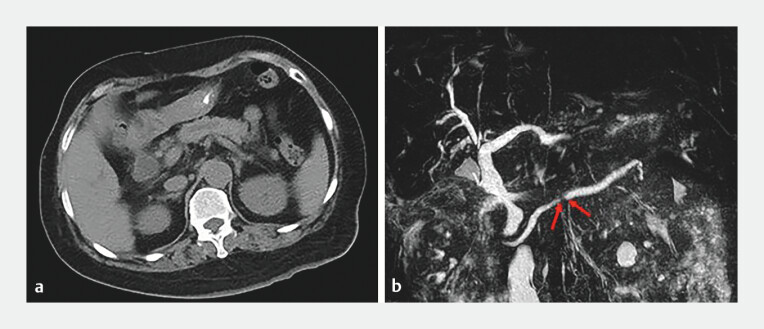
Preoperative imaging showed a main pancreatic duct (MPD) stricture in the body and following upstream duct dilation, with absence of masses and calculi. **a** Computed tomography. **b** Magnetic resonance cholangiopancreatography.

Therefore, endoscopic retrograde pancreatography (ERP) was planned. After successful pancreatic cannulation, repeated attempts to advance the guidewire through the stricture failed ( Fig. 2 ). Then, a new peroral pancreatoscope (eyeMax, 9 Fr; Micro-Tech, Nanjing, China) was inserted. A pinhole-like concentric stricture with smooth mucosa, without protrusions, friability or tumor vessels was found, suggestive of a benign nature 1 ( Fig. 3 ). Despite guidewire manipulation under direct vision, the stricture was so narrow that only a 0.025-inch ultra-fine guidewire (Jagwire; Boston Scientific, Marlborough, Massachusetts, USA) was able to pass through (a 0.035-inch failed) ( Fig. 4 ). In addition, the stricture was so tight that neither a sphincterotome nor dilation bougies were able to cross, and complete guidewire blockage prevented passage of contrast agent. A Soehendra stent retriever (Cook Medical, Bloomington, Indiana, USA) was then used to negotiate across the stricture. Finally, a 5-Fr × 11-cm pancreatic plastic stent (Zimmon; Cook Medical) was successfully placed ( Video 1 , Fig. 5 ). At the six-month follow-up, the patient remained asymptomatic and free of disease progression.

**Fig. 2:**
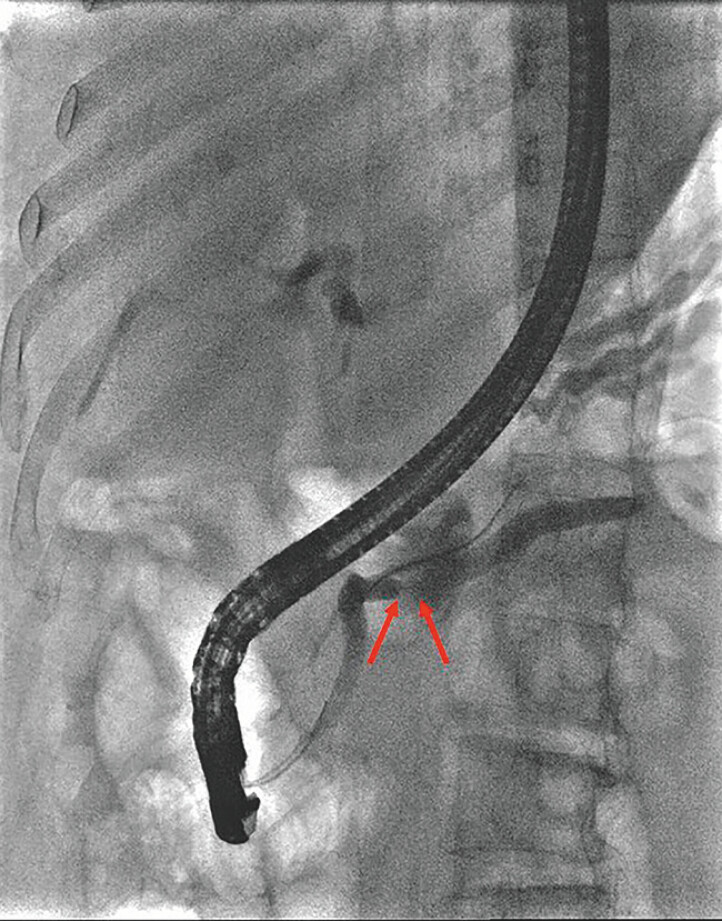
Contrast imaging demonstrated a stricture of the pancreatic duct in the pancreatic body.

**Fig. 3:**
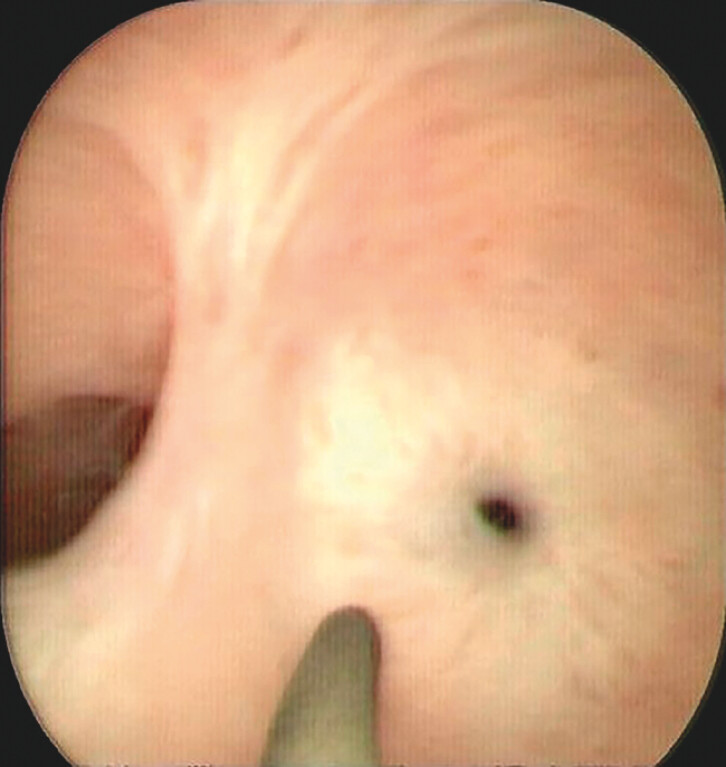
Endoscopic appearance of MPD stricture by new peroral pancreatoscopy.

**Fig. 4:**
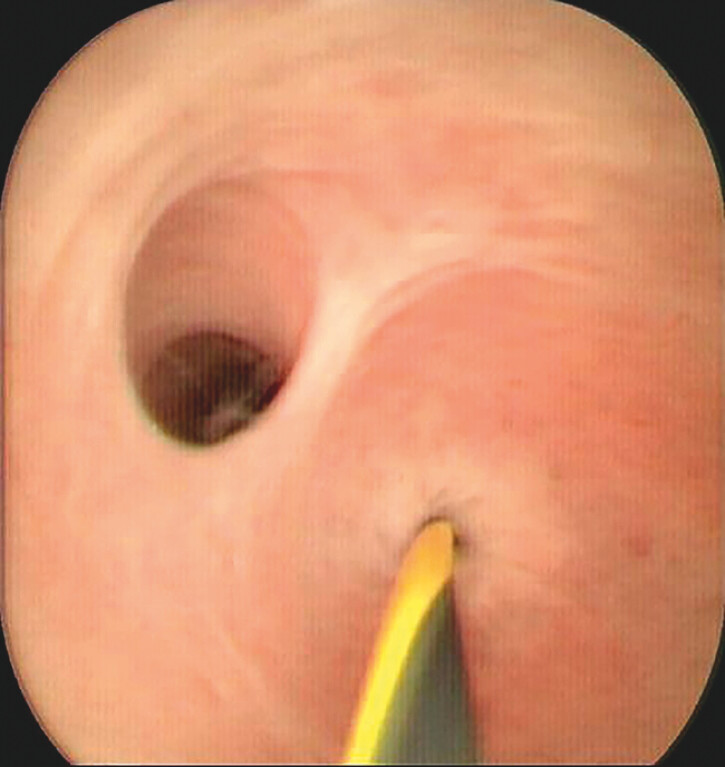
Pancreatoscopy-guided precision guidewire placement.

**Fig. 5:**
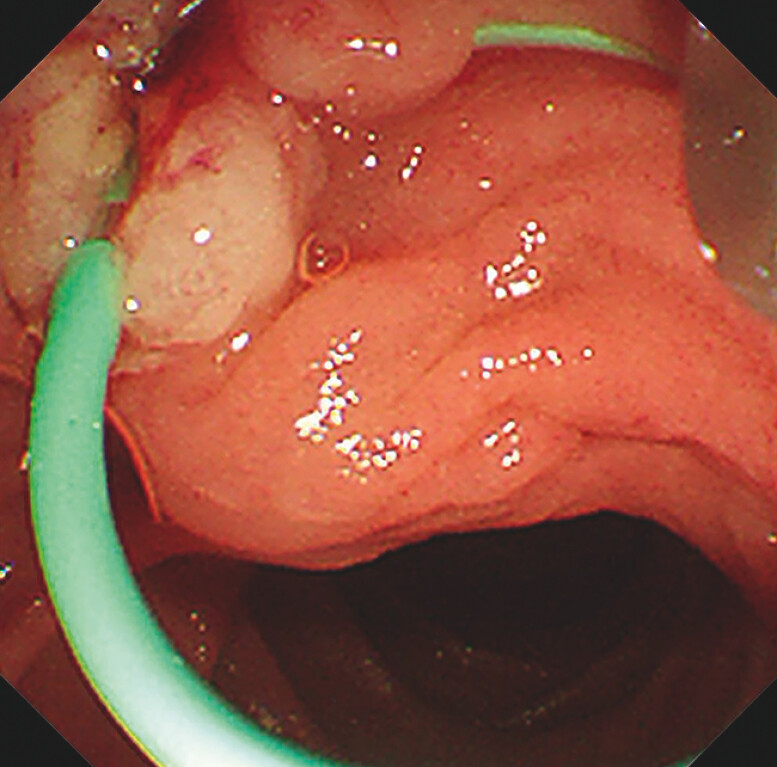
Successful placement of a pancreatic stent.

New peroral pancreatoscopy for direct diagnosis and precise guidewire placement in indeterminate pancreatic duct stricture.Video 1

Non-pancreatitis benign MPD stricture is rare and its differentiation from malignancy is challenging. Similar to cholangioscopy, peroral pancreatoscopy is useful in the diagnosis of an indeterminate MPD stricture by providing direct visualization and targeted biopsy. Endoscopic ultrasound (EUS)-assisted rendezvous often serves as an important rescue procedure when conventional ERP fails 2 3 . In our case, pancreatoscopy was an effective alternative method. To our best knowledge, this is the first case of pancreatoscopy-guided precision guidewire placement and stenting for non-pancreatitis benign MPD stricture.

Endoscopy_UCTN_Code_TTT_1AR_2AI

## Notes

Endoscopy E-Videos https://eref.thieme.de/e-videos
*E-Videos* is an open access online section of the journal *Endoscopy* , reporting on interesting cases and new techniques in gastroenterological endoscopy. All papers include a high-quality video and are published with a Creative Commons CC-BY license. Endoscopy E-Videos qualify for HINARI discounts and waivers and eligibility is automatically checked during the submission process. We grant 100% waivers to articles whose corresponding authors are based in Group A countries and 50% waivers to those who are based in Group B countries as classified by Research4Life (see: https://www.research4life.org/access/eligibility/ ). This section has its own submission website at https://mc.manuscriptcentral.com/e-videos .
